# The complete mitochondrial genome sequence of the hawk moth, *Theretra oldenlandiae* (Lepidoptera: Sphingidae)

**DOI:** 10.1080/23802359.2020.1719929

**Published:** 2020-01-31

**Authors:** Xu Wang, Hao Zhang, Wei-Ying Qu, Yi-Xin Huang

**Affiliations:** Anhui Provincial Key Laboratory of the Conservation and Exploitation of Biological Resources, Key Laboratory of Biotic Environment and Ecological Safety in Anhui Province, College of Life Sciences, Anhui Normal University, Wuhu, Anhui, China

**Keywords:** Mitogenome, mtDNA, phylogenetic relationship

## Abstract

In this study, we analyzed the complete mitochondrial genome sequence of the hawk moth, *Theretra oldenlandiae*. The complete mitogenome sequence of *T. oldenlandiae* was observed to be a circular molecule 15,312 bp long and consisting of 13 protein-coding genes (PCG), 2 ribosomal RNA (rRNA) genes, and 22 transfer RNA (tRNA) genes (GenBank accession number MN885801). The nucleotide composition is biased toward adenine and thymine (80.0% A + T). The A + T-rich region was found between *rrnS* and *trnM*, and this entire region was 423 bp long.

The hawk moth, *Theretra oldenlandiae* (Fabricius) is distributed in southern and eastern Asia, and is an important insect pest that feeds on agricultural crops and ornamental plants (Sambath 2011; Rafi et al. 2014; Rougerie et al. 2014). Despite its economic importance, the mitogenome sequence of *T. oldenlandiae* so far remains unknown. Therefore, we sequenced the complete mitochondrial DNA genome of *T. oldenlandiae* to provide more comprehensive data for this species and also for its relationship within the family Sphingidae.

Larva of *T. oldenlandiae* was collected from the campus of Anhui Normal University (N31°20′9.37′′ and E118°22′9.14′′), Anhui, China in August 2019 and deposited in the Entomological Museum, College of Life Sciences, Anhui Normal University (AHNU) under the accession no. AHWH20190322. The complete mitochondrial genome of *T. oldenlandiae* was determined by using next-generation sequencing (NGS).

The *T. oldenlandiae* mitochondrial genome is 15,312 bp (GenBank accession MN885801) in length with a total A + T content of 80.0% that is heavily biased toward the A and T nucleotides. It encodes the complete set of 37 genes, which are usually found in animal mitogenomes. In the mitogenome of *T. oldenlandiae*, a total of 26 bp overlaps have been found at nine gene junctions. The mitogenome is loose and has a total of 131 bp intergenic sequences without the putative A + T-rich region. The intergenic sequences are at 12 locations ranging from 1 to 54 bp, with the longest one located between *trnQ* and *nad2*. The A + T-rich region of the *T. oldenlandiae* is 423 bp long and located between the *rrnS* and *trnM* genes. The A + T content of this region is 95.5%.

All 22 tRNA genes usually found in the mitogenomes of insects are present in *T. oldenlandiae*. The nucleotide length of tRNA genes ranges from 64 bp (*trnC*) to 71 bp (*trnK*), and A + T content ranges from 70.4% (*trnK*) to 92.5% (*trnE*). These two rRNA genes have been identified on the N-strand in the *T. oldenlandiae* mitogenome.

We analyzed the nucleotide sequences of PCGs using the maximum-likelihood (ML) method to understand the phylogenetic relationship of *T. oldenlandiae* with other Sphingids. The mitogenome sequence of *Saturnia boisduvalii* (GenBank accession number MF034742) was used as an outgroup. The result shows that *T. oldenlandiae* belongs to the family Sphingidae and is clustered into a branch of *Theretra* (Figure 1).

**Figure 1.**
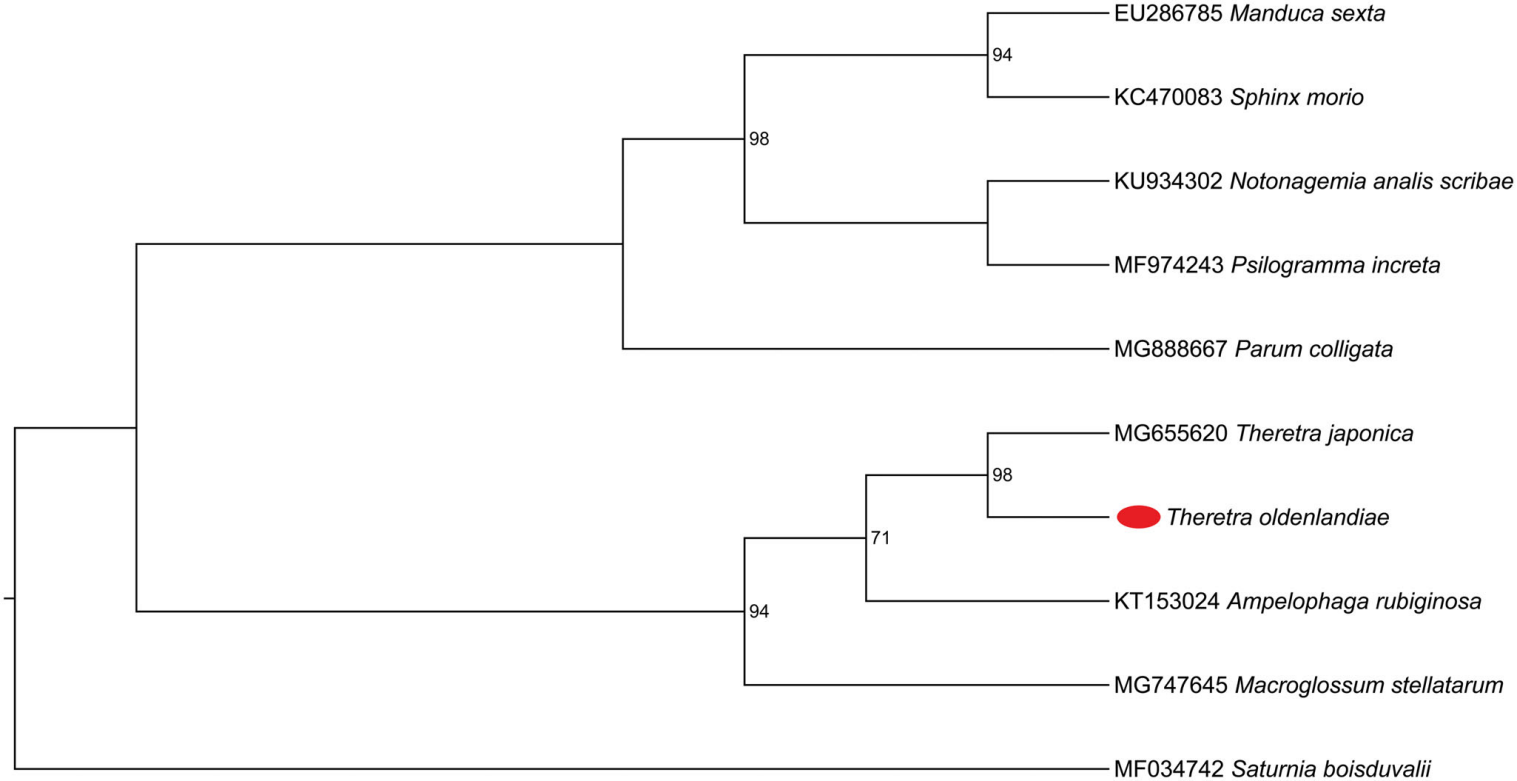
The maximum-likelihood (ML) phylogenetic tree of *Theretra oldenlandiae* and other Sphingids. The numbers beside the nodes are percentages of 1000 bootstrap values. Alphanumeric terms indicate the GenBank accession numbers.
